# Empathic competencies in violent offenders^☆^

**DOI:** 10.1016/j.psychres.2013.08.027

**Published:** 2013-12-30

**Authors:** Eva-Maria Seidel, Daniela Melitta Pfabigan, Katinka Keckeis, Anna Maria Wucherer, Thomas Jahn, Claus Lamm, Birgit Derntl

**Affiliations:** a Social, Cognitive and Affective Neuroscience Unit, Department of Basic Psychological Research and Research Methods, Faculty of Psychology, University of Vienna, Liebiggasse 5, 1010 Vienna, Austria; b Prison for mentally disordered offenders (Section 21/2 of the Austrian Penal Code), Justizanstalt Wien-Mittersteig, Vienna, Austria; c Department of Psychiatry, Psychotherapy and Psychosomatics, Medical School, RWTH Aachen University, Aachen, Germany

**Keywords:** Affect, Social, Skin conductance, Criminals, Violence

## Abstract

Violent offending has often been associated with a lack of empathy, but experimental investigations are rare. The present study aimed at clarifying whether violent offenders show a general empathy deficit or specific deficits regarding the separate subcomponents. To this end, we assessed three core components of empathy (emotion recognition, perspective taking, affective responsiveness) as well as skin conductance response (SCR) in a sample of 30 male violent offenders and 30 healthy male controls. Data analysis revealed reduced accuracy in violent offenders compared to healthy controls only in emotion recognition, and that a high number of violent assaults was associated with decreased accuracy in perspective taking for angry scenes. SCR data showed reduced physiological responses in the offender group specifically for fear and disgust stimuli during emotion recognition and perspective taking. In addition, higher psychopathy scores in the violent offender group were associated with reduced accuracy in affective responsiveness. This is the first study to show that mainly emotion recognition is deficient in violent offenders whereas the other components of empathy are rather unaffected. This divergent impact of violent offending on the subcomponents of empathy suggests that all three empathy components can be targeted by therapeutic interventions separately.

## 1 Introduction

Identifying and responding to emotional states of other people is a crucial skill for successful social interaction. It has been hypothesized that aggressive behavior may result from a deficit in adequately recognizing and responding to social cues, mainly distress-related cues (Blair, 2001, 2005).

Most studies investigating empathic competencies in violent offenders focused on *emotion recognition*. Hoaken et al. (2007) reported a general deficit in this ability in violent offenders compared to both non-violent offenders and controls. However, a meta-analysis of 20 studies also revealed no general impairment but a specific deficit in the recognition of fearful, sad and surprised expressions in antisocial offenders (Marsh and Blair, 2008). Comparing sex and non-violent offenders, Gery et al. (2009) showed deficient recognition of disgusted, angry and fearful faces in sex offenders only. A recent study also observed specific deficits in recognizing facial expressions of sadness, fear, disgust and anger in violent and non-violent offenders compared to intelligence-matched controls (Robinson et al., 2012). Dolan and Fullam (2006) reported deficits in the recognition of sad, happy and surprised faces in violent offenders. Interestingly, these authors also found a negative correlation between recognition accuracy for sad faces and the psychopathy score.

Considering the effects of psychopathy on previous results, findings are rather mixed. This may be due to different tasks applied in previous studies as well as differences in sample characteristics and comparison groups. While some studies report a general emotion recognition deficit of psychopathic offenders (Hastings et al., 2008) others observed a specific deficit in recognizing fear only (Blair et al., 2004; Iria and Barbosa, 2009). Adding to the complexity, others could not find any deficits in emotion recognition (Glass and Newman, 2006; Book et al., 2007) or observed deficits in disgust recognition together with an even better accuracy for recognizing angry faces in psychopathic offenders (Kosson et al., 2002). Habel et al. (2002) showed deficits in recognizing both happy and sad faces in a sample of male psychopaths but also reported a positive correlation between recognition accuracy and the affective component of psychopathy. The authors interpreted this finding in terms of a necessary ability to read others in order to deceive and manipulate.

However, emotion recognition is only one component of empathy (Decety and Jackson, 2004). Marshall et al. (1995) and Marshall and Marshall (2011) postulate a staged model of empathy based on research in violent offenders: The first stage includes recognizing the emotional state of other. Next, this observation of the other's emotion is enhanced by taking the perspective of the other person. In the third stage, emotion recognition and perspective taking enable the observer to feel an emotional response. Especially with regard to distress cues (also see the violence-inhibition model as reviewed by Blair (2005)), Marshall and Marshall postulated that if the observer has a positive or neutral relationship with the other person, he/she will be able to take the perspective of the other (stage 2) and show a compassionate response (stage 3). If the observer has a hostile relationship or is overwhelmed by the distress, two possibilities that have been assumed in the case of violent offenders, then the observer does not progress to the next stages.

Previous studies on *perspective taking* in antisocial populations used different tasks and methodologies tapping either cognitive or emotional perspective taking. Sex offenders seem to show deficits in cognitive perspective taking as measured with so-called higher order cognitive theory of mind tasks (Castellino et al., 2011) as well as emotional perspective taking (Elsegood and Duff, 2010) measured via the reading the mind in the eyes task (Baron-Cohen et al., 2001). Regarding psychopathy, Dolan and Fullam (2004) reported no differences in cognitive perspective taking between psychopathic and non-psychopathic offenders and controls. Shamay-Tsoory et al. (2010) replicated these null findings for cognitive perspective taking in psychopathic offenders, but observed deficits in emotional perspective taking. However, Richell et al. (2003) failed to show any differences between psychopathic offenders and controls in emotional perspective taking using the reading the mind in the eyes task.

Despite some inconsistencies of previous results regarding emotion recognition and perspective taking the current evidence suggests that especially psychopathic offenders are largely able to understand others' emotions on a cognitive level.

The so-called “emotion paradox” (Lorenz and Newman, 2002) states that psychopathic offenders seem to recognize emotions but do not show a compassionate response. Previous studies have investigated this reduced *affective responsiveness* with skin conductance measures (for a meta-analysis see Lorber (2004)). Herpertz et al. (2001) reported emotional hyporesponsiveness in skin conductance to both positive and negative pictures in psychopathic offenders whereas offenders with borderline personality disorder did not differ from healthy controls. Notably, all three groups showed comparable self-reported emotional responses, similar to results from Habel et al. (2002) using a mood induction paradigm. Applying an anger induction to a sample of offenders with antisocial personality disorder, Lobbestael et al. (2009) reported cardiovascular hyporeactivity compared to controls but again no differences in self-reported anger levels. Testing non-psychopathic offender groups with and without antisocial personality disorder, Wahlund et al. (2010) also observed lower skin conductance responses (SCRs) but also reduced self-reported emotional responses to negative pictures. These studies demonstrated that not only psychopathy but violent offending in general seems to be associated with reduced affective responsiveness.

The present study is the first attempt to directly test the three stage model of empathy in violent offenders compared to age and intelligence matched healthy controls. Though somewhat inconsistent, previous evidence suggests that violent offenders show specific impairments in more cognitive components of empathy, i.e. emotion recognition and perspective taking, but seem to display pronounced deficits in affective responsiveness. There is no previous study testing all three components in the same sample and additionally, differences in sample and task characteristics make it difficult to infer whether and how the three components of empathy are interrelated in violent samples. Therefore, we applied a well-validated task (Derntl et al., 2009a, 2009b; Seidel et al., 2012) tapping emotion recognition, perspective taking, and affective responsiveness separately. This design enabled us to clarify potential associations and interactions between different empathy components in violent offenders. Furthermore, we combined those accuracy tasks with a physiological measure of arousal, the SCR. Biological measures, such as SCR, are preferable compared to self-report data as they may be less prone to biases, such as socially desirable responding or other types of deception.

The inclusion of specific empathy-focused treatment approaches is a feature of most contemporary violent offender treatment programs (cf. Day et al., 2010). An additional exploratory aim of this study was to examine the effects of an empathy focused group intervention on empathic competencies measured by our task.

We predicted that there is no general deficit in recognizing emotions in other people's faces or in taking the perspective of others but rather an emotion-specific impairment as shown in most previous studies (e.g., Dolan and Fullam, 2006; Gery et al., 2009; Robinson et al., 2012). Moreover, we hypothesized that violent offenders show a significantly decreased affective and physiological response to emotional stimuli. We further expected that deficits in the affective response would be most pronounced in offenders with high psychopathy scores.

## 2 Methods

### 2.1 Sample

Thirty male incarcerated violent offenders and 30 healthy males matched for age, education and intelligence (see Table 1) participated in this study. The study was carried out in accordance with the latest version of the Declaration of Helsinki and approved by the Institutional Review Board of the Ministry of Justice (enforcement agency, JVD). All participants took part on a voluntary basis, gave written informed consent and received 10€/h for participation. It was ensured to the offender group that participating in this study will have no consequences for any juridical or other decisions regarding their prison status.

The offender group was recruited from a local correctional facility consisting of 30 violent offenders who on average had spent more than 2000 days in prison already (mean: 2220.86 (902.94)). Most offenders (*n*=22) were diagnosed with cluster B personality disorders according to DSM-IV criteria and some with disorders of sexual preference (pedophilia [*n*=4], exhibitionism [*n*=2], unspecified [*n*=2]). Some offenders had a history of alcohol (*n*=10) or drug dependence (*n*=3). All psychiatric diagnoses have been confirmed using the SCID I and II interview. All offenders have been rated on the Psychopathy Checklist Revised (PCL-R, Hare, 1991) by trained and experienced psychologists (mean: 21.5 (7.23), mean factor I: 8.73 (3.34), mean factor II: 10.79 (3.89)). Hartmann et al. (2001) argued for a cut-off value of 25 for European studies, which has been applied e.g., by Berger et al. (2012). However, a recent study used a lower cut-off score of 21 (Domes et al., 2013). Our sample can therefore on average be considered a medium to high-scoring sample.

At the time of testing some offenders (*n*=17) were taking the prescribed psychopharmaceutical medication (antidepressant [*n*=3], neuroleptic [*n*=8], antidepressants+neuroleptic [6]). Comparing medicated vs. unmedicated offenders did not reveal any differences (see Section 3.7). Furthermore, we divided the group into sexually violent offenders and non-sexually violent offenders. This did not reveal any significant differences for accuracy or SCR data (all *p*-values >0.177).

Also, some offenders (*n*=11) participated in a standardized empathy-focused group treatment once during their term of imprisonment. During 10 sessions the group treatment included mainly the discussion of moral dilemmas and perspective switching exercises (see Lind et al., 2010). In order to explore the effects of this empathy-focused group therapy with comparable sample sizes, we randomly selected 15 controls and compared those to 11 treated and 19 untreated offenders. All three groups did not differ regarding age (*F*(2, 44)=0.78, *P*=0.925) or intelligence (*F*(2, 44)=0.18, *P*=0.982) on a matrices test.

The non-violent control group consisted of 30 healthy adults with no history of psychiatric or neurological illness as well as no substance abuse in themselves and in their first degree relatives. The control group was recruited by advertisements. Sample characteristics are shown in Table 1.

### 2.2 Tasks

For the present study we modified a set of three tasks tapping emotion recognition (Derntl et al., 2009a), perspective taking and affective responsiveness (Derntl et al., 2009b). The response format has been changed from a dichotomous choice between two emotion categories/faces to a forced choice format containing six emotional categories in all three tasks. This is a main improvement of the previous tasks, which has been suggested by the authors in recent publications (e.g., Seidel et al., 2012). Choosing between six different categories is considerably increasing the difficulty of the task compared to choosing from two alternatives only. In addition to helping to avoid ceiling effects in high-ability groups, this response format might reflect reality more accurately.

**Fig. 1:**
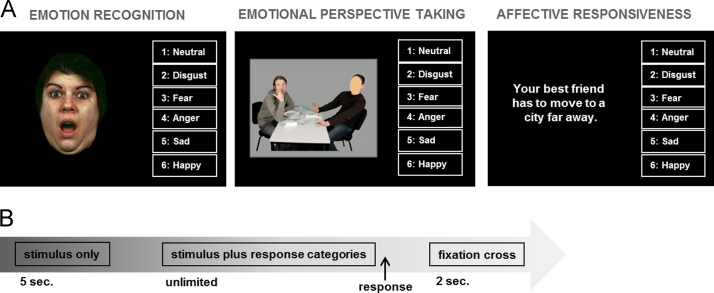
Visualization of the empathy paradigm. (A) Stimulus plus response categories per task and (B) Timeline per trial.

Stimulus material was presented on a 15.4 in. laptop monitor (Dell Latitude) using E-Prime 2.0 software (Psychology Software Tools, Inc., Sharpsburg, PA, USA). For illustration of the stimulus material and response format see Fig. 1. For all tasks, each stimulus is only presented once and all materials are gender-balanced. Stimuli were presented for 5 s and remained on the screen together with response categories until a response was selected (see Fig. 1B). The interstimulus interval was 2 s.

#### 2.2.1 Emotion recognition

Thirty-six colored photos of Caucasian faces (taken from a standardized stimulus set, cf. Gur et al., 2002) depicting five basic emotions (happiness, sadness, anger, fear, disgust) and neutral expressions were presented. Participants had to determine the correct emotion by selecting from six emotional categories. Responses were given on a laptop keyboard via button press. Stimuli were presented for 5 s, followed by response categories which were presented until a response was given.

#### 2.2.2 Emotional perspective taking

Participants viewed 57 pictures each presented for 5 s depicting scenes showing two Caucasians involved in social interaction thereby portraying five basic emotions and neutral scenes. In a validation study, stimuli were rated by 30 healthy adults and only those stimuli correctly identified by over 70% of the sample were included in the published task (see Derntl et al., 2009b). This resulted in 10 stimuli for disgust, happy and neutral as well as nine stimuli for anger, fear and sad. The face of one person was masked and participants were asked to infer the corresponding emotional expression of the masked face that would fit the emotional situation. Stimuli were presented for 5 s, followed by response categories which were presented until a response was given. Responses were made by selecting from six emotional categories.

#### 2.2.3 Affective responsiveness

Participants read 55 short written sentences describing real-life situations, which are likely to induce basic emotions (10 happy, 8 sad, 10 anger, 9 fear, 8 disgust) and 10 situations that were emotionally neutral. Participants were asked to imagine how they would feel if they were experiencing those situations. Stimuli were presented for 5 s, followed by response categories which were presented until a response was given. Responses were made by selecting from six emotional categories.

### 2.3 Empathy questionnaires and intelligence measure

All participants completed the German version of the Interpersonal Reactivity Index (IRI, Davis, 1983) as a self-report measure of dispositional empathic traits. In addition, they completed a Rasch homogeneous version of Raven's standard progressive matrices (SPM; Van der Ven and Ellis, 2000) tapping reasoning, which is considered to be a valid indicator for non-verbal intelligence.

### 2.4 Skin conductance data acquisition

Inmates were tested in a quiet room in the prison; controls were tested in a laboratory room at the Faculty of Psychology, University of Vienna. Both groups were tested in the morning (8 am to 11 am). Room temperature was held constant between 23 and 24 °C in both settings. Participants were seated in a comfortable chair with their non-dominant forearm placed on a cushion on the table in front of them. After electrode attachment, participants positioned themselves comfortably and were asked to avoid any unnecessary movements during measurement. The whole experiment took about 90 min in total. Note that, additional experimental tasks performed before the present experiment (outside of the scope of this paper) will be presented elsewhere. Skin conductance data were recorded using an 8-channel bioamplifier (Mobi8-BP; TMSI B. V., Enschede, The Netherlands) with a 24 bit A/D conversion rate. Time-locked stimulus onsets were acquired using PortiLab 2.0 software (TMSI B. V., Enschede, The Netherlands). The acquisition of unfiltered raw skin conductance data was guaranteed using a custom-specific skin conductance sensor. Two flat Ag/AgCl electrodes were placed at the medial phalanges of the index and the ring fingers of the non-dominant hand. Prior to electrode application, the skin was cleaned using curd soap. Skin conductance data were sampled at 1024 Hz for digital storage. The experiment started after a waiting period of approximately 10 min after electrode application to ensure stable skin conductance levels. Meanwhile, participants filled in the IRI questionnaire.

### 2.5 Statistical analysis

#### 2.5.1 Behavioral data

Statistical analyses were performed using SPSS Statistics 19.0 and level of significance was set at *P*=0.05.

Accuracy and self-report data were analyzed using repeated-measures ANOVAs with emotion/scale as within-subject factor and group as between-subject factor. Statistical tests involving the emotion factor employed the Huynh–Feldt correction if the sphericity assumption was not met. Estimates of effect size (partial *η*²) are listed for significant differences. Group differences regarding age, intelligence and education were assessed using *t*-tests. Correlations between psychopathy scores, number of violent assaults, number of days in prison and total accuracy in the empathy tasks were computed using the Spearman coefficient (note that one offender was considered an outlier as he had spent more than 6000 days in prison and therefore this person was removed from this correlation analysis). Also, we correlated corresponding SCR and accuracy data.

Social situations involving anger or fear seem to be highly relevant in the context of violent offending. Therefore, we performed correlations between anger and fear processing and the number of violent assaults.

#### 2.5.2 Skin conductance data

Data pre-processing and analysis steps were carried out using Matlab 7.9.0 (The MathWorks, Inc., Natick, MA) and the Matlab-based toolbox Ledalab^®^ V3.4.2 (Leipzig, Germany) which is available online (www.ledalab.de). Skin conductance data were downsampled to 10.24 Hz. Subsequent removal of artifact-afflicted trials and data smoothing were carried out using Ledalab. Continuous decomposition analysis (CDA; Benedek and Kaernbach, 2010a) was performed to disentangle phasic components from the tonic activity based on standard deconvolution. This method returns the skin conductance level as a continuous measure of tonic electrodermal activity, as well as the phasic driver underlying skin conductance data as a continuous measure of phasic electrodermal activity. Due to technical errors (artifacts or non-responding) SCR data of three control participants as well as three offenders had to be excluded from analysis.

Stimulus-driven changes in skin conductance were analyzed within a response window of 5 s starting at stimulus onset. We relied on the so-called SCR value, which represents the average phasic driver within the response window. This score is the result of a multi-step deconvolution approach applied to SCR data. The method is based upon a physiological model of the general shape of the SCR (Benedek and Kaernbach, 2010b). SCR data were outlier corrected per group based on box-plots provided by SPSS.

## 3 Results

### 3.1 Emotion recognition

#### 3.1.1 Behavioral data

**Fig. 2:**
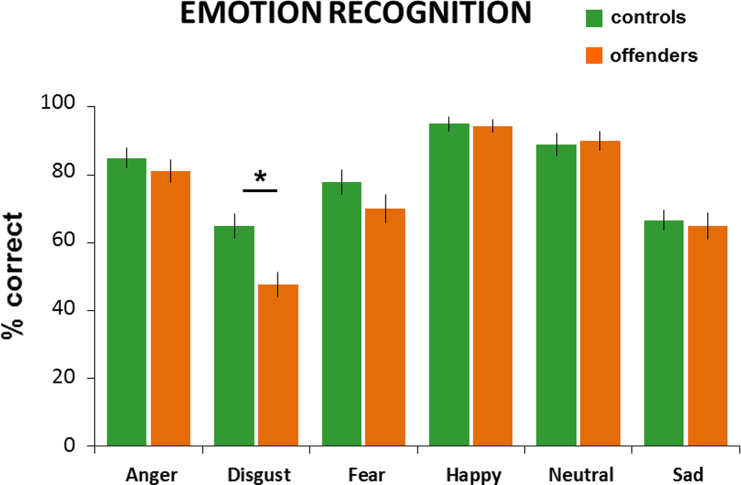
Mean accuracy values (plus standard error of mean) per emotion for both groups.

The repeated-measures ANOVA revealed a significant effect of emotion (*F*(4.715, 273.488)=43.182, *P*<0.001, partial *η*²=0.427) with highest accuracy for happy faces and lowest for disgust. Also, a significant effect of group occurred (*F*(1, 58)=5.191, *P*=0.026, partial *η*²=0.082) with controls outperforming offenders (see Fig. 2). Moreover, we observed a significant emotion by group interaction (*F*(4.715, 273.488)=2.297, *P*=0.049, partial *η*²=0.038). Planned comparisons showed that controls outperformed offenders for disgust only (*t*(58)=3.362, *P*=0.001), while for all other emotions no significant difference emerged (all *P*-values>0.164). See Table 2 for means (S.D.) of both groups.

#### 3.1.2 SCR

**Fig. 3:**
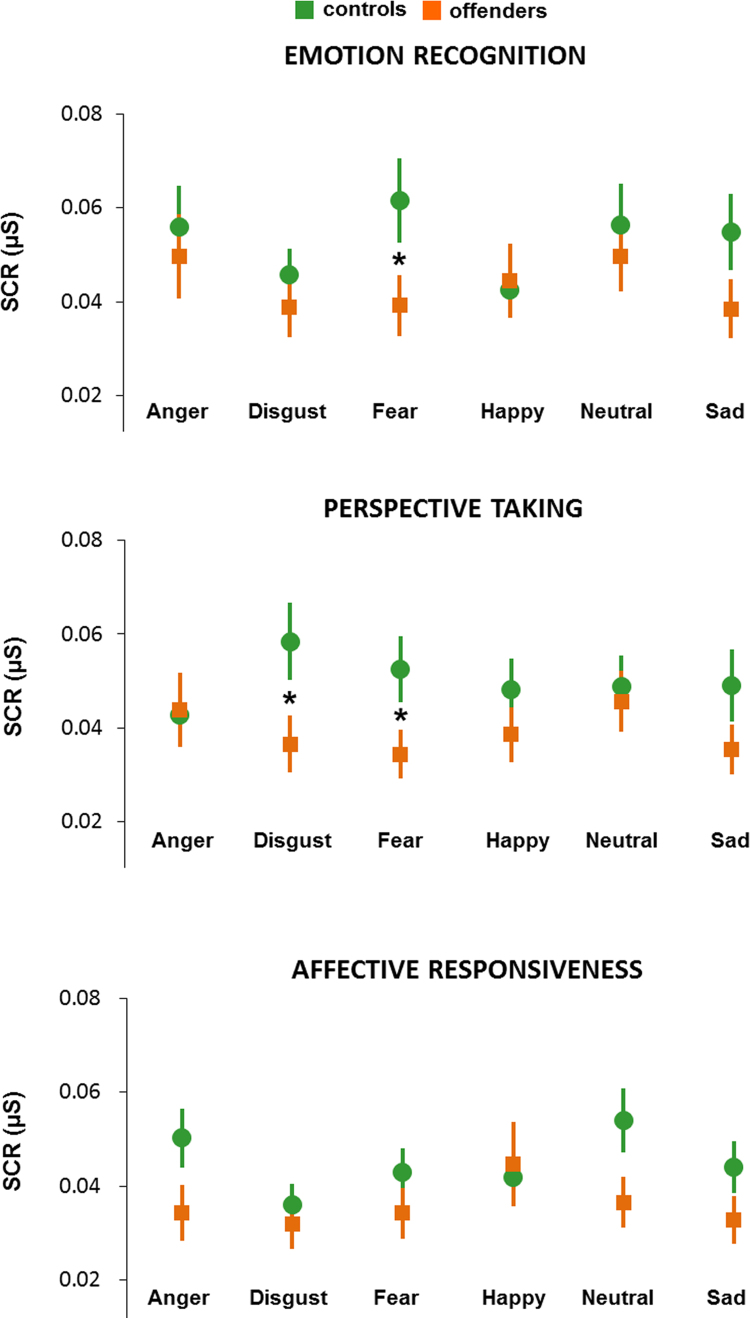
Mean SCR values per emotion (plus standard error of mean) for all three paradigms.

We observed a significant effect of emotion (*F*(5, 260)=2.912, *P*=0.014, partial *η*²=0.053) with strongest SCR to angry and neutral faces. There was no significant main effect of group (*F*(1, 52)=1.008, *P*=0.320) but a significant emotion by group interaction occurred (*F*(5, 260)=2.444, *P*=0.035; partial *η*²=0.045). Planned comparisons revealed significantly diminished SCR in the offender compared to the control group only for fearful faces (*t*(52)=2.026, *P*=0.048). No other comparison reached significance (all *P*-values>0.116). See Fig. 3a for illustration.

### 3.2 Emotional perspective taking

#### 3.2.1 Behavioral data

Accuracy data revealed a significant effect of emotion (*F*(4.382, 254.160)=22.696, *P*<0.001, partial *η*²=0.281) with highest accuracy for happy scenes and lowest accuracy for fearful scenes. No significant effect of group (*F*(1, 58)=1.672, *P*=0.201) and no significant emotion by group interaction occurred (*F*(4.382, 254.160)=0.964, *P*=0.433). See Table 2 for means (S.D.) of both groups.

#### 3.2.2 SCR

There was no significant emotion effect (*F*(5, 260)=1.065, *P*=0.380) and no significant effect of group (*F*(1, 52)=1.653, *P*=0.204). However, we observed a significant emotion by group interaction on the SCR data (*F*(5, 260)=3.975, *P*=0.002, partial *η*²=0.071). Planned comparisons revealed significantly weaker SCR in offenders compared to controls for disgusting (*t*(52)=2.130, *P*=0.038) and fearful scenes (*t*(52)=2.065, *P*=0.044) (see Fig. 3).

### 3.3 Affective responsiveness

#### 3.3.1 Behavioral data

A significant effect of emotion occurred (*F*(4.518, 262.058)=14.226, *P*<0.001, partial *η*²=0.197) with highest accuracy for happy sentences and lowest accuracy for disgusting sentences. No significant effect of group (*F*(1, 58)=1.720, *P*=0.195) and no significant emotion by group interaction (*F*(4.518, 262.058)=1.364, *P*=0.242) was observed. See Table 2 for means (S.D.) of both groups.

#### 3.3.2 SCR

SCR data revealed a significant effect of emotion (*F*(5, 260)=4.344, *P*=0.001, partial *η*²=0.077) with strongest SCR in response to neutral sentences and weakest to fearful and sad sentences. There was no significant group effect (*F*(1, 52)=1.457, *P*=0.233) but a significant emotion by group interaction (*F*(5, 260)=3.787, *P*=0.003, partial *η*²=0.068). Planned comparisons showed only a trend for group differences in response to neutral (*t*(52)=1.987, *P*=0.052) and angry sentences (*t*(52)=1.1818, *P*=0.075) with a trend for weaker SCR in offenders compared to controls (see Fig. 3).

### 3.4 Correlation analyses

In the offender group we observed a significant correlation between emotion recognition accuracy and emotional perspective taking accuracy (*r*=0.465, *P*=0.010). In the control group, a significant correlation between accuracy in the emotional perspective taking and affective responsiveness task (*r*=0.440, *P*=0.015) emerged. All other correlations between tasks did not reach significance (all *P*-values>0.142). Moreover, no significant association between SCR and corresponding accuracy data (all *P*-values>0.040) was observed.

Correlating total accuracy and number of violent assaults for each task did not reveal any significant associations (all *P*-values>0.107). Correlating total accuracy scores of affective responsiveness with PCL I scores showed a significant negative association (*r*=−0.427, *P*=0.019). PCL I scores did not correlate with total SCR data (all *P*-values>0.455). PCL II scores were not correlated to total accuracy or total SCR data (all *P*-values>0.181). Number of days in prison was not correlated to total accuracy or total SCR data (all *P*-values>0.75).

Correlating accuracy scores of anger and fear items of each task with the number of violent assaults showed a significantly negative association with anger recognition in the emotional perspective taking task only (*r*=−0.427, *P*=0.019).

### 3.5 IRI

Self-report data of the IRI showed significant differences between ratings on the four subscales (*F*(3, 174)=54.334, *P*<0.001. partial *η*²=0.484), with highest ratings for perspective taking and lowest for personal distress. There was a significant effect of group (*F*(1, 58)=6.909, *P*=0.011, partial *η*²=0.106) with offenders reporting higher values than controls. Moreover, a significant scale by group interaction occurred (*F*(3, 174)=9.047, *P*<0.001, partial *η*²=0.135). Post-hoc planned comparisons revealed a significant group difference for personal distress only (*t*(58)=4.742, *P*=<0.001) with offenders reporting more personal distress than controls. This was correlated with accuracy for disgust in the emotion recognition task (*r*=−0.422, *P*=0.006). All other correlations remained non-significant (*P*-values>0.108).

### 3.6 Effects of therapy

Analyzing the effects of empathy-focused group therapy revealed a significant effect of group for emotion recognition (*F*(2, 42)=3.482, *P*=0.040, partial *η*²=0.142) and emotional perspective taking (*F*(2, 42)=4.509, *P*=0.017, partial *η*²=0.177) but none for affective responsiveness (*F*(2, 42)=1.454, *P*=0.245). Post-hoc comparisons showed that performance of the untreated offender group differed significantly from that of controls (emotion recognition: *P*=0.030, perspective taking: *P*=0.030) and from the performance of the treated group (emotion recognition: *P*=0.036, perspective taking: *P*=0.009). As expected, the untreated group showed lower accuracy compared to the treated group and the control group, which did not differ (emotion recognition: *P*=0.908, perspective taking: *P*=0.519). As described for the whole sample, we observed a significant emotion effect (emotion recognition: *F*(5,210)=28.795, *P*<0.001, partial *η*²=0.407; perspective taking: *F*(5,210)=18.678, *P*<0.001, partial *η*²=0.308; affective responsiveness: *F*(4.602, 193.268)=9.245, *P*<0.001). However, no interaction effects occurred (all *P*-values>0.168). Also, skin conductance data and self-report data (IRI) were not affected by the empathy-focused group therapy (all *P*-values>0.163).

### 3.7 Effects of medication

Comparing medicated offenders (*n*=17) with unmedicated offenders (*n*=13) did not reveal significant group differences for accuracy data (emotion recognition: *F*(1, 28)=0.720, *P*=0.403; emotional perspective taking: *F*(1, 28)=0.033, *P*=0.858; affective responsiveness: *F*(1, 28)=1.035, *P*=0.318) or SCR data (emotion recognition: *F*(1,25)=0.438, *P*=0.514; emotional perspective taking: *F*(1, 25)=0.015, *P*=0.904; affective responsiveness: *F*(1, 25)=0.298, *P*=0.590).

## 4 Discussion

Violence and aggressive behavior have often been associated with a lack of empathy or empathic responses. The present study aimed at clarifying which core component of empathy is altered in violent offenders with varying degrees of psychopathy compared to non-offender controls. To this end, we applied three behavioral tasks tapping emotion recognition, emotional perspective taking and affective responsiveness and recorded SCR data. There were three principle findings: First, on the behavioral level, we observed significantly reduced accuracy in emotion recognition, mainly for disgust, in violent offenders compared to healthy controls. There were no group differences regarding accuracy in emotional perspective taking and affective responsiveness. However, we observed a negative association of psychopathy and accuracy in the affective responsiveness task. Second, SCR data showed reduced physiological responses in the offender group compared to controls for fear (emotion recognition and emotional perspective taking) and disgust stimuli (emotional perspective taking). Third, exploratory analysis of the effect of an empathy-focused group therapy showed better emotion recognition and emotional perspective taking abilities in treated offenders.

Our findings suggest that violent offenders are impaired in recognizing facial emotional expressions and in particular expressions of disgust. This finding is in accordance with previous reports of specific rather than general emotion recognition deficits (Gery et al., 2009; Robinson et al., 2012) in offender samples. Recent evidence has associated disgust with human morality (Pizarro et al., 2011) and inhibition of moral violations, such as incest (Lieberman et al., 2007; Fessler and Navarrete, 2004). Nevertheless, disgust was the emotion which was most difficult to recognize also for the control group. Both groups frequently mistook disgust for sadness, anger or fear. The deficient recognition abilities of violent offenders may be more obvious for expressions that are more difficult to recognize in general. For example, happiness is generally easier to infer from a facial expression, therefore, we did not expect group differences for this emotion. There is only one previous study reporting deficits in happiness recognition (Dolan and Fullam, 2006).

However, when confronted with more complex social stimuli, such as social scenes in the emotional perspective taking task, performance of offenders and controls did not differ. This task seemed to be more difficult for both groups compared to emotion recognition. However, we observed a negative association of number of violent assaults and accuracy in perspective taking for angry scenes. This correlative result suggests that offenders with many violent assaults are more impaired in taking the perspective of other people in situations where anger is the prevalent emotion. This has intuitive appeal taking into account that anger and rage play a crucial role in violent delinquency. The anger stimuli in our perspective taking task depicted mainly social scenes of threat or social conflict. The participant was required to take the perspective of the aggressor (with a masked face) and recognize that this person is feeling anger. Interestingly, those offenders with supposedly most experience with such scenes in the role of the aggressor had most difficulties in identifying the correct emotion.

As expected according to the emotion paradox (Lorenz and Newman, 2002), we did not observe an association between psychopathy and our two more cognitive tasks, emotion recognition and emotional perspective taking. However, with increasing interpersonal and affective psychopathy traits (i.e. the core psychopathic personality) accuracy in affective responsiveness decreased. Additionally, with increasing number of violent assaults, as an indicator of impulsive, antisocial traits, we observed a decrease in emotional perspective taking. This divergent effect of indicators of psychopathy vs. impulsivity regarding more affective and more cognitive components of empathy is highly informative regarding specific treatment approaches.

SCR data revealed emotion specific arousal increases in controls but no such modulation in the offender group. Fearful facial expressions seem to be a particularly strong, biologically relevant facial cue (Whalen et al., 1998), eliciting high arousal. Also, the biologically most relevant emotional contents (fear and disgust) in the more complex social scene stimuli used in the emotional perspective taking task elicited a significant physiological response in controls. Our data are in accordance with previous reports of reduced SCR in violent offenders in general and not only those with high psychopathic traits (e.g., Wahlund et al., 2010). Hence, the offender group seemed to correctly recognize fear and disgust scenes but did not show a comparable physiological response.

To our knowledge, this is the first study testing the three stages of empathic accuracy described in the model put forward by Marshall et al. (1995) and Marshall and Marshall (2011) in the same sample. We observed a significant association between accuracy in emotion recognition and emotional perspective taking as well as between accuracy in the emotional perspective taking and affective responsiveness task in the whole sample. Our data do not fully support the staged model. Despite the emotion recognition deficit, which reflects a basic impairment on the first stage, we could not observe deficient perspective taking (stage 2) or affective responsiveness (stage 3) in the offender sample. In accordance with the divergent correlation results, our findings suggest that the different components of empathy are working rather independently than based on each other. Applying the same battery of tasks in different psychiatric samples (Derntl et al., 2012) showed that all samples were impaired in affective responsiveness, i.e. the third stage, although there was no overall impairment in the first two stages. Therefore, our current and also previous results are more in line with theories on empathy based on social neuroscience research. For example, Decety and Lamm (2006) proposed that empathy involves both bottom-up (e.g., affective sharing) as well as top-down (e.g., perspective taking) mechanisms that interact with each other but are not based on each other.

However, one should consider that lacking group differences in emotional perspective taking and affective responsiveness in our study may also be due to floor and ceiling effects, respectively. The perspective taking task seemed complicated for controls as well, whereas the affective responsiveness task seemed to be too easy for both groups, which may be considered a limitation of this study. Furthermore, our SCR data suggest emotion-specific reduced physiological responsiveness, which can also be seen as one indicator of reduced affective responsiveness.

Exploring the effects of a standardized empathy-focused group treatment in our offender sample, we found that both more cognitive empathy components, i.e. emotion recognition and perspective taking, can be improved. Although the preliminary effects of treatment were more pronounced for the emotional perspective taking task, our results suggest that there is an active transfer to basic emotion recognition. However, we cannot determine how long the effects last, as we do not have information on when each inmate participated. The group treatment particularly focused on mentalizing abilities by e.g., discussion of moral dilemmas and perspective switching exercises (see Lind et al., 2010). Therefore, it is not surprising that there was neither a behavioral nor SCR difference between untreated and treated offenders for affective responsiveness. As this study was not designed as a treatment study, we do not have a baseline measure of empathic competency of the treated group. Day et al. (2010) point out that up to now there is limited evidence on whether empathy-focused interventions can improve empathy and more importantly whether those interventions will also reduce recidivism in the long run. Based upon our promising but restricted results it would be highly interesting to examine the effects of a therapy program tailored to target both cognitive and affective components of empathy in longitudinal studies and evaluating outcome performance with our three paradigms.

Despite several interesting findings the study has some limitations that have to be taken into account in interpreting the results. We think that in particular, the affective responsiveness paradigm could be improved by using more naturalistic interpersonal understanding tasks, like the empathy paradigms applied by Regenbogen et al. (2012) or Zaki et al. (2009). Most studies investigating psychopathy or violent offending focused on male inmates (Wynn et al., 2012). Up to now there is no study on empathic competencies in female offender groups, except for some self-report questionnaire results (for a review see Rogstad and Rogers (2008)). Given well-replicated gender differences in empathy in healthy samples (e.g., Derntl et al., 2010; Rueckert and Naybar, 2008; Schulte-Rüther et al., 2008), studying female offender groups could offer relevant insights on the interaction of empathy, gender and offending.

## Figures and Tables

**Table 1:** Demographic data (mean and S.D. in parentheses).

	**Offender group (*****n*****=30)**	**Control group (*****n*****=30)**	***t***	**P**
Age	35.6 (12.5)	34.8 (10.2)	0.260	0.796
Education (years)	11.37 (2.19)	12.30 (1.66)	1.851	0.069
SPM raw-score	23.50 (5.50)	23.10 (3.54)	0.335	0.739

*Note*: SPM=Standard Progressive Matrices.

**Table 2:** Accuracy data for both groups (mean percent correct and S.D.).

	**ANGER**	**DISGUST**	**FEAR**	**HAPPY**	**NEUTRAL**	**SAD**
Emotion recognition						
Offender group	81 (18)	48 (20)	70 (23)	94 (11)	90 (15)	65 (21)
Control group	85 (16)	65 (20)	78 (20)	95 (12)	89 (18)	67 (16)

Perspective taking						
Offender group	76 (20)	67 (12)	59 (13)	85 (14)	78 (22)	63 (21)
Control group	82 (15)	68 (16)	61 (14)	85 (11)	77 (16)	71 (19)

Affective responsiveness						
Offender group	88 (15)	83 (15)	93 (11)	97 (4)	92 (14)	81 (19)
Control group	89 (14)	72 (24)	89 (18)	97 (6)	89 (16)	81 (18)
